# Genomic Investigation of Population Structure in a Secretive, Semi‐Fossorial Snake (Colubridae: *Tropidoclonion lineatum*) Across the Great Plains

**DOI:** 10.1002/ece3.74431

**Published:** 2026-09-28

**Authors:** Taylor Probst, Cameron D. Siler, Drew R. Davis

**Affiliations:** ^1^ School of Biological Sciences University of Oklahoma Norman Oklahoma USA; ^2^ Oklahoma Biological Survey University of Oklahoma Norman Oklahoma USA; ^3^ Department of Biology Eastern New Mexico University Portales New Mexico USA; ^4^ Department of Integrative Biology The University of Texas at Austin Austin Texas USA

**Keywords:** fossorial snake, population genetics, taxonomy

## Abstract

With advances in genomic technologies, it has become increasingly feasible to generate large numbers of loci to address population genetic and phylogeographic questions. Nevertheless, many widespread and relatively abundant species remain unsampled using molecular approaches. The lined snake ( *Tropidoclonion lineatum* ), a semi‐fossorial snake distributed across the Great Plains, is one such example. Despite the historical recognition of multiple subspecies based on noted variation in morphological characters, no formal molecular investigation of population structure has been conducted to date due to a paucity of vouchered genetic samples. Here, we present the first genomic assessment of range‐wide population structure in *T. lineatum* using genome‐wide single‐nucleotide polymorphisms (SNPs) generated via triple‐enzyme restriction site‐associated DNA (3RAD) sequencing. Our results indicate largely continuous gene flow across the Great Plains, questioning the validity of the recognized subspecies. In contrast, populations at the northern range edge in South Dakota exhibit relative genetic homogeneity and modest differentiation from more southern populations, potentially reflecting recent isolation or increased genetic drift in peripheral habitats. Given the species' endangered status in South Dakota, these findings highlight the conservation importance of range‐edge populations while underscoring the need for denser, range‐wide sampling—particularly in putatively disjunct regions—to fully characterize genetic diversity in *T. lineatum* .

## Graphical abstract

Here we provide the first examination of population structure and genetic variation within a secretive semi‐fossorial snake ( *Tropidoclonion lineatum* ), widely distributed across the Great Plains. In our evaluation, we assess the validity of the four historically described subspecies and identify a new potentially isolated population. Our findings reveal widespread gene flow, with conservation implications for the state‐endangered isolated population.

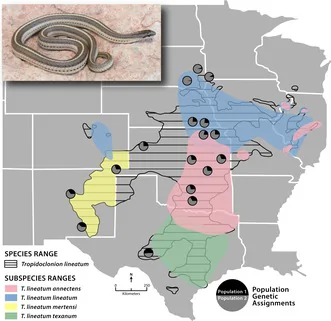

## 1 Introduction

Fossoriality, a life history strategy defined by subterranean burrowing (Cyriac and Kodandaramaiah 2018), has evolved multiple times within squamate reptiles (lizards and snakes; Anelli et al. 2024; Bergmann and Morinaga 2019; Brandley et al. 2008; Greer 1991) and is thought to be the ancestral condition of snakes (Da Silva et al. 2018; Longrich et al. 2012; Martill et al. 2015). Among fossorial snakes, there exists a spectrum of specialization, ranging from highly derived forms such as the scolecophidians, characterized by microphthalmy, increased cranial ossification, and miniaturization (Da Silva et al. 2018; Kley 2006; Miralles et al. 2018), to lineages lacking obvious morphological modifications, such as *Diadophis* and *Storeria* (Willson and Dorcas 2004). The secretive nature of fossorial snakes complicates their detection (Durso et al. 2011; Matthias et al. 2021), thereby hindering studies of their natural history, population abundance, and evolution (Miralles et al. 2018; Turner 1977; Willson and Dorcas 2004). As a result, biases exist in herpetofaunal survey data and museum collections, resulting in many common and widespread fossorial snake species lacking basic assessments of population and phylogeographic structure, despite growing evidence that many fossorial squamates harbor unrecognized cryptic diversity (Fontanella et al. 2008; Graboski et al. 2023; Parham and Papenfuss 2009; Thomas and Hedges 2007). The lined snake ( *Tropidoclonion lineatum* ), a small, fossorial snake in a monotypic genus that is distributed across much of the Great Plains in the United States, is one such example (Stebbins 2003).

Lined snakes spend most of the time underground (Fogell 2010; Schultz and Caven 2021) and are proficient burrowers in loose, moist soils, where they forage for earthworms and other small invertebrates (Stebbins 2003). Although *T. lineatum* occurs primarily in grasslands, open woods, and flood plains below 1500 m (Fogell 2010; Stebbins 2003), records exist of individuals found above 2000 m, including one from Torrance County, New Mexico, at approximately 2810 m elevation (Hathcock et al. 2018). The species is distributed across much of the central United States from south‐central Texas to the northern limits of its range in southwestern Minnesota and South Dakota, where it is considered a species of special concern and state endangered, respectively (Minnesota Department of Natural Resources 2013; South Dakota Game, Fish, and Parks 2024). Despite its broad geographic range, relatively few tissue samples from *T. lineatum* are available in museum collections to our knowledge (e.g., no tissue samples are available in public institutions from Colorado populations), and the species has only been included in a small number of higher‐level molecular phylogenetic analyses (Alfaro and Arnold 2001; Deepak et al. 2022; McVay and Carstens 2013; Zaher et al. 2019). Consequently, intraspecific structure and population‐level genetic variation within the species remain poorly understood.

Despite a lack of molecular sampling, five subspecies of *Tropidoclonion lineatum* have been described based primarily on minor differences in caudal and ventral scale counts: *T. l. lineatum* (Hallowell 1856; Ramsey 1953), *T. l. iowae* (Call 1891), *T. l. mertensi* (Smith 1965), *T. l. texanum* (Ramsey 1953), and *T. l. annectens* (Ramsey 1953). Shortly after its description, *T. l. iowae* was synonymized with *T. l. lineatum* (Smith and Smith 1963; Stejneger 1891) due to a lack of diagnostic character differences. The remaining subspecies are not widely recognized in published literature (Nicholson 2025; Stebbins 2003), as the ranges proposed by Ramsey (1953) and Smith and Smith (1963) overlap extensively with broad areas of intergradation among the subspecies (Figure 1). Yet, many reptiles and amphibians distributed across the Great Plains exhibit substantial population divergence and genetic structure (Finger et al. 2022; Fontanella et al. 2008; Waraniak et al. 2019), therefore similar patterns across the broad distribution of *T. lineatum* may be expected. Thus, the taxonomic validity of subspecies of *T. lineatum* warrants further investigation through modern molecular methods.

**FIGURE 1:**
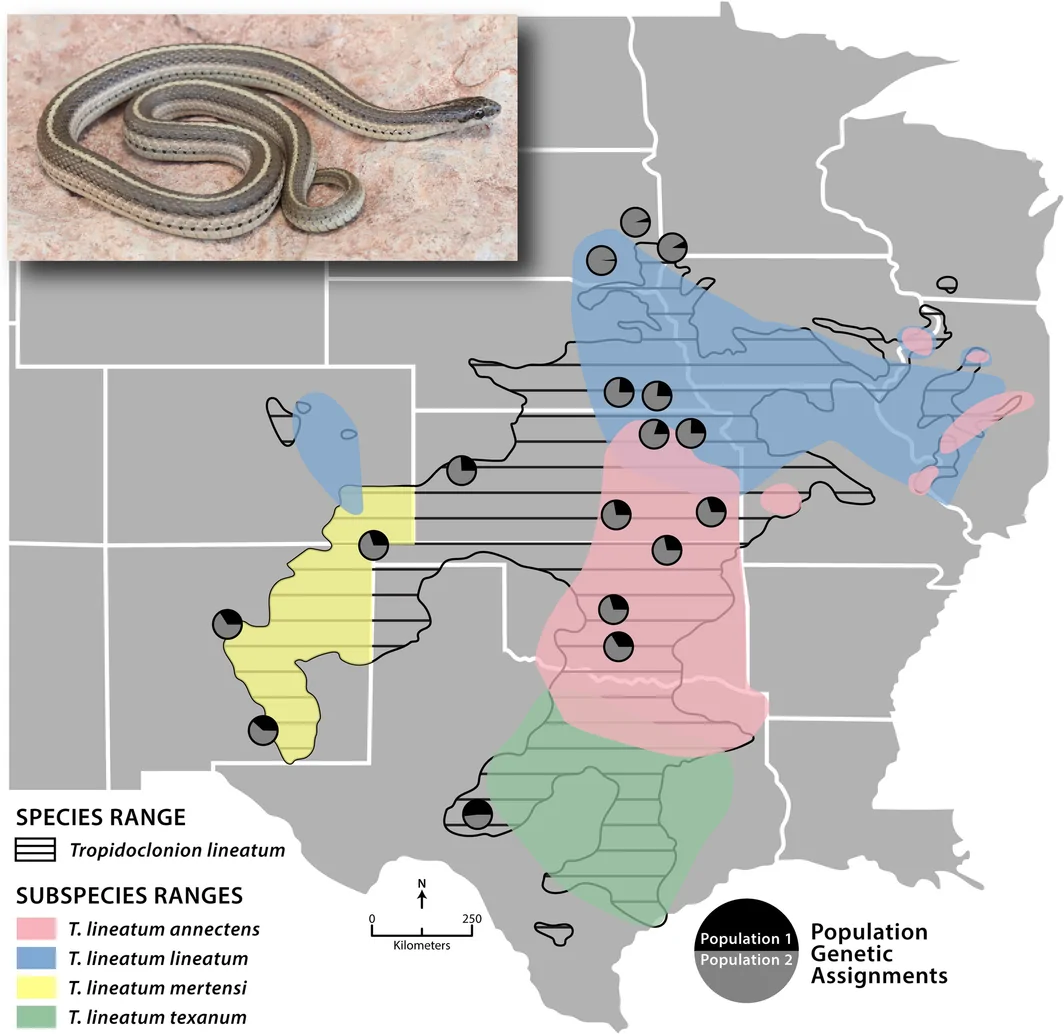
Distribution of *Tropidoclonion lineatum* samples used in this study mapped onto the current range (shown in horizontal dashed lines) obtained from the Center of North American Herpetology (https://www.cnah.org/). The proposed ranges of each subspecies are given, drawn based on maps from Ramsey (1953) and Smith and Smith (1963), with the range of *T. l. mertensi* estimated from descriptions of the northwestern range edge in Smith (1965). While Ramsey (1953) had proposed Illinois to only include *T. l. lineatum*, Smith and Smith (1963) noted that specimens bordering the Mississippi river are best treated as intergrades of *T. l. lineatum* and *T. l. annectens*, with populations further west resembling *T. l. annectens*. To reflect these distinctions, we overlayed Smith and Smith's (1963) map onto Ramsey's (1953) range. Samples are represented as a pie chart, with proportions corresponding to population assignments as inferred by STRUCTURE (see Figure 2B). Due to redundancy in proportions and the larger sample size of the James River population, the population is represented by a single pie chart.

In addition to subspecies designations as hypotheses of geographic differentiation, populations at the periphery of a species' range may exhibit substantial genetic divergence as differential selective regimes reduce effective gene flow from core populations while promoting local adaptation (Angert et al. 2020; Bontrager et al. 2021; Sexton et al. 2009). The recent discovery of a seemingly disjunct lined snake population along the James River in South Dakota may represent one such population (Davis and Farkas 2018). Within South Dakota, the lined snake was thought to occur only in habitats along the Big Sioux River (Backlund 2000; Ballinger et al. 2000; Kiesow 2006), until recent surveys located a population along the James River in Hutchinson County (Davis and Farkas 2018; Kiesow and Davis 2020). This population along the James River occupies an area of only ca. 4000 ha and appears to be isolated from populations of lined snakes along the Big Sioux River, as no other lined snakes have been detected further south along the James River in Yankton County, nor have they been detected along the Vermillion River to the east. The lack of verifiable records between the James and Big Sioux rivers, combined with the limited dispersal ability of these small, fossorial snakes may suggest that this James River population is an isolated, potentially unique lineage of lined snakes.

In this study, we employ genome‐wide single‐nucleotide polymorphism (SNP) data generated using triple‐enzyme restriction site‐associated DNA (3RAD) sequencing to carry out a preliminary assessment of range‐wide population structure in the lined snake, estimate effective population size of the James River population, and assess gene flow with the James River population to the core population. Our results provide the first examination of population genomic structure in *Tropidoclonion* and we discuss implications of the findings for taxonomy and conservation status of this enigmatic, widespread, fossorial snake.

## 2 Materials and Methods

We obtained 36 tissue samples (liver, tail clips, and blood) for *T. lineatum* , representing seven of the 12 states in which the species is found (Appendix 1). Unfortunately, we were unable to locate any lined snake tissue samples from Iowa, Illinois, Missouri, Arkansas, or Colorado. Total genomic DNA was extracted in the Core Genomics Lab at the Sam Noble Oklahoma Museum of Natural History (University of Oklahoma) using Qiagen DNAeasy Blood & Tissue Kits (Qiagen, Hilden, Germany). Extracts were quantified using a Qubit, and aliquots were sent to the Oklahoma Medical Research Foundation (OMRF, Oklahoma City, OK) Sequencing Facility for genomic library preparation and genotyping using 3RAD (3‐enzyme Restriction site‐Associated DNA sequencing) sequencing methodologies (Bayona‐Vásquez et al. 2019). Samples were digested with NheI‐HF, EcoRI‐HF, and XbaI enzymes, size‐selected for approximately 150 bp, and sequenced on an Illumina NovaSeq X Plus.

De‐multiplexing, adapter trimming, and read filtering were performed in iPyrad v0.9.105 (Eaton and Overcast 2020). Filtering followed the default iPyrad parameters, with zero mismatches allowed in the barcodes, applying a strict adapter filter (2), with reads requiring base calls to exceed a minimum quality score of 20. Individuals for which fewer than 250,000 raw reads were recovered due to sequencing failure were removed from subsequent analyses because insufficient sequencing coverage prevented the recovery of a sufficient number of meaningful loci during clustering and assembly. Reads were then assembled into de novo loci, with the clustering threshold set to 0.90 as determined to be appropriate for population genetics (Eaton and Overcast 2019). Although inappropriate clustering thresholds can bias genotype inference by splitting orthologs at high thresholds and clustering paralogs at low thresholds (Ilut et al. 2014; Mastretta‐Yanes et al. 2015), genetic diversity and differentiation are generally robust to moderate variation in this parameter (Gargiulo et al. 2021). To reduce these risks, sequencing depth was checked to ensure that depth was > 10 for a cluster, and that the hetero_est was between 0.001–0.05 (Mastretta‐Yanes et al. 2015), with the error rate ~0.001 to reflect the average error rate of Illumina reads (Stoler and Nekrutenko 2021) (Appendix 2). For assembled loci to be included in the final assembly, we used default parameters specifying that the loci needed to be shared across a minimum of four samples with the max number of Ns (ambiguous/unknown base calls) not exceeding a proportion of 0.05.

To examine genetic structure and cluster assignment, we first carried out Bayesian model‐based clustering using STRUCTURE v2.3.4 (Pritchard et al. 2000) through iPyrad's API, testing values of *K* from 1 to 7 under an admixture model with correlated allele frequencies. We specified mincov = 0.9 (retaining SNPs shared across at least 90% of samples), minmap = 0.5 (requires 50% coverage in specified groups), and ran 10 replicates per *K* value with 100,000 MCMC iterations, discarding the first 50,000 as burn‐in. To visualize individual ancestry proportions, we generated bar plots of STRUCTURE results. The second method we used for examining genetic clusters was principal components analysis (PCA) through ipyrad's built‐in analysis tools. Like our STRUCTURE runs, we specified mincov = 0.9, minmap = 0.5, using *k*‐means clustering with *k* set to 2 clusters (reflecting the optimal numbers of clusters found in STRUCTURE analysis) for 1000 iterations. Results were then visualized through scatterplots.

To explore phylogenetic relationships within our dataset, we used three phylogeny‐based approaches: phylogenetic networks, maximum likelihood (ML) inference, and a multi‐species coalescent (MSC) inference. Because a single bifurcating tree cannot fully capture the genealogical relationships within a population (Leaché et al. 2023), phylogenetic networks are useful for visualizing reticulate patterns of evolution and identifying admixed samples between populations. (Blair and Ané 2020). Phylogenetic networks were constructed from the concatenated SNP dataset excluding non‐variable sites using the SplitsTree App v6.7.6 (Huson and Bryant 2024) with the NeighborNet algorithm (Bryant and Moulton 2004) and uncorrected P‐distances.

Several limitations can complicate phylogenetic inference from RADseq SNP datasets, including allelic dropout (Arnold et al. 2013), acquisition bias (Leaché et al. 2015; Leaché and Oaks 2017), and concatenation of loci (Edwards et al. 2016; Leaché and Oaks 2017). In particular, allelic dropout can have a significant impact on outgroup selection, as the number of orthologous loci shared with the ingroup decreases as phylogenetic distance increases (Arnold et al. 2013; Gautier et al. 2013). While an optimal outgroup would be *Regina grahamii* or another Thamnophiine snake (Alfaro and Arnold 2001), no such taxa were included within our dataset. As an alternative, we attempted to root the phylogeny using two species of Philippine snakes sequenced on the same plate, *Cyclocorus lineatus* and *Calamaria gervaisii* . However, even under lenient filtering thresholds, too few orthologous loci were recovered, and the phylogenetic distances between the outgroups and the ingroup were too great (Huelsenbeck et al. 2002; Swofford et al. 1996) to permit confident rooting of the ingroup. Accordingly, we chose to visualize relationships using unrooted trees. In the absence of a root, evolutionary polarity cannot be assessed and reciprocal monophyly cannot be established in a strict rooted sense; nevertheless, unrooted trees still permit evaluation of whether particular groups of samples form well‐supported, distinct genetic clusters that would be consistent with monophyly under at least one possible rooting (Wilkinson et al. 2007).

We generated a Maximum Likelihood (ML) tree using RAxML v8.2.12 (Stamatakis 2014) under the GTR+Γ (GTRGAMMA) substitution model. To reduce the effect of missing data, we filtered the SNP matrix to retain only biallelic sites shared across 85% of samples through VCFtools v0.1.17 (Danecek et al. 2011), specifying ‐‐min‐alleles 2, ‐‐max‐alleles 2, and ‐‐max‐missing 0.85. We then performed 100 rapid bootstrap replicates, followed by 10 independent ML searches from random starting trees using rapid hill‐climbing. To visualize relationships among individuals using an MSC approach, we constructed an MSC tree with Tetrad v0.9.14 as implemented in iPyrad (Eaton and Overcast 2020), specifying nquartets = 1e6 to analyze all possible quartets, with 1000 bootstrap replicates to assess support. Tetrad uses the SVDquartets algorithm (Chifman and Kubatko 2014), which estimates quartet relationships from SNP data across all possible four‐taxon subsets and then assembles these quartet trees into a species tree.

To assess signatures of isolation by distance (Wright 1943), we used the ape v5.8.1 package (Paradis and Schliep 2019) in R (R Core Team 2024) to calculate raw (uncorrected) pairwise genetic distances between individuals from the filtered 85%, biallelic SNP dataset. These were then plotted against pairwise geographic distances as calculated using the geosphere v1.6.5 package (Hijmans et al. 2017). A Mantel test was used to assess correlation between genetic and geographic distances as implemented in the R package vegan v2.7.3 (Oksanen et al. 2013).

To estimate the contemporary effective population size (*N*_e_) for the James River population in Hutchinson County, South Dakota, we used the linkage disequilibrium (LD) method implemented in N_e_Estimator v2.1 (Do et al. 2014) on the filtered 85%, biallelic SNP dataset (excluding sample TNHC 111755 due to high proportions of missing data). Because physical linkage between SNPs of close genomic proximity can bias *N*_e_ downwards (Waples et al. 2016), we performed an additional analysis of the 85%, biallelic SNP dataset, manually filtering to retain the first SNP of every locus (retaining 10,293 SNPs). We assumed random mating and examined minor allele frequency thresholds of *P*_crit_ = 0.05, 0.02, and 0.01 (unfiltered) to assess sensitivity to the inclusion of rare alleles (Waples and Do 2010). Confidence intervals (CI) were calculated using both the parametric and jackknife methods across loci, and final interpretations were based on the *P*_crit_ = 0.02 threshold. To compare heterozygosity of the James River population (*n* = 15) to the core population, we calculated observed heterozygosity using the HIERFSTAT v0.5‐11 package (Goudet 2005) on both the filtered 85%, biallelic SNP dataset and single‐SNP‐per‐locus dataset (as used for calculating *N*_e_). To avoid confounding genetic signals from other samples at the range edge, our core population was restricted to samples within Kansas, Oklahoma, and Nebraska (*n* = 11). To determine the level of gene flow and identify potential population isolation in the James River population, we used the Python package scikit‐allel v1.3.13 (Miles et al. 2024) to calculate *F*‐statistics, implementing the Weir and Cockerham (1984) estimator similarly on both datasets as specified above for *N*_e_ and *H*_o_.

## 3 Results

Our sequencing efforts resulted in the recovery of 132,499 loci and 684,738 single‐nucleotide polymorphisms (SNPs) from 36 lined snake samples following filtering. Unfortunately, four samples failed to meet quality requirements and were thus removed from the dataset for downstream analyses (Appendix 1). Of the 23,264 SNPs retained after filtering for STRUCTURE and PCA, 8483 were unlinked and used to infer population structure. STRUCTURE analyses identified an optimal clustering of *K* = 2 according to the Evanno ∆*K* method (Evanno et al. 2005). STRUCTURE analysis revealed weak population structure along a latitudinal gradient (Figures 1 and 2B). Individuals from northern populations primarily cluster with genetic group 1, whereas southern individuals show a higher proportion of genetic assignment to group 2. The gradual change in allele frequency across the range suggests weak genetic differentiation rather than sharply defined population boundaries. Furthermore, this latitudinal genetic cline is particularly evident along PC1, which explains 11.6% of the total variation (Figure 2A). Northern samples have negative PC1 scores (left side of the plot), while samples further south progressively shift toward positive PC1 scores (right side), reflecting gradual genetic differentiation along the latitudinal gradient.

**FIGURE 2:**
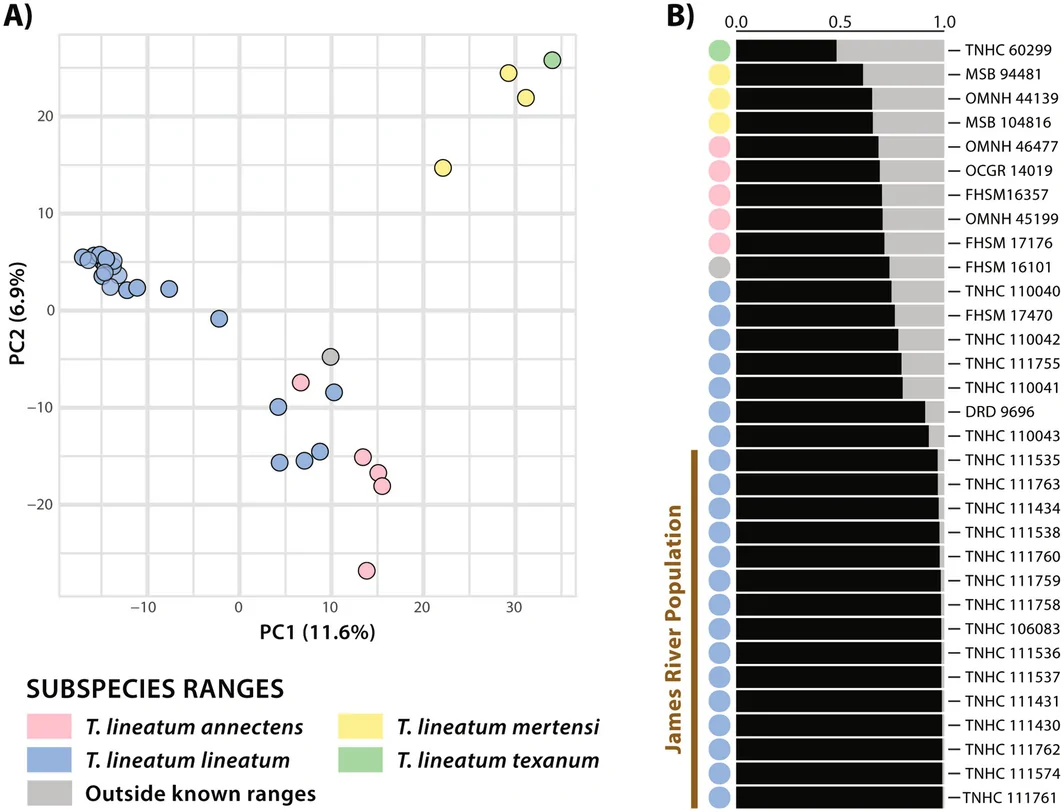
Population structure of 32 *Tropidoclonion lineatum* individuals inferred from 8483 SNPs using STRUCTURE. (A) Principal components analysis (PCA) of genetic distance between samples based on the filtered SNP dataset. The relative contribution of each principal component (labeled as a percentage) is given next to axis labels. Samples are colored according to their respective subspecies (B) Bar plot showing individual membership proportions for Δ*K* = 2, with black representing Cluster 1 and gray representing Cluster 2. Sample subspecific designation and museum accession numbers are shown below each bar, with colors coded according to the legend provided to the right of the PCA plot.

Filtering to retain biallelic SNPs shared across 85% of samples resulted in 58,091 SNPs in our final matrix. Phylogenetic network analysis of *T. lineatum* supports the latitudinal genetic cline inferred from our clustering analyses (Figure 3A), as most samples cluster to geographically proximate localities with intermediate individuals positioned in between. For example, samples from Nebraska (TNHC 110040–110042, FHSM 17470) are positioned between those of South Dakota (North, *T. l. lineatum*) and Kansas (South, *T. l. annectens*). One exception is sample TNHC 111755 (Hutchinson County, SD), which is recovered as a cluster to an Oklahoma sample. Given the high proportion of missing data in this individual (80.67% of the final concatenated loci dataset), this placement may be artifactual despite filtering thresholds. Consistent with this interpretation, TNHC 111755 also shows anomalous clustering in STRUCTURE, PCA, and ML, and MSC analyses.

**FIGURE 3:**
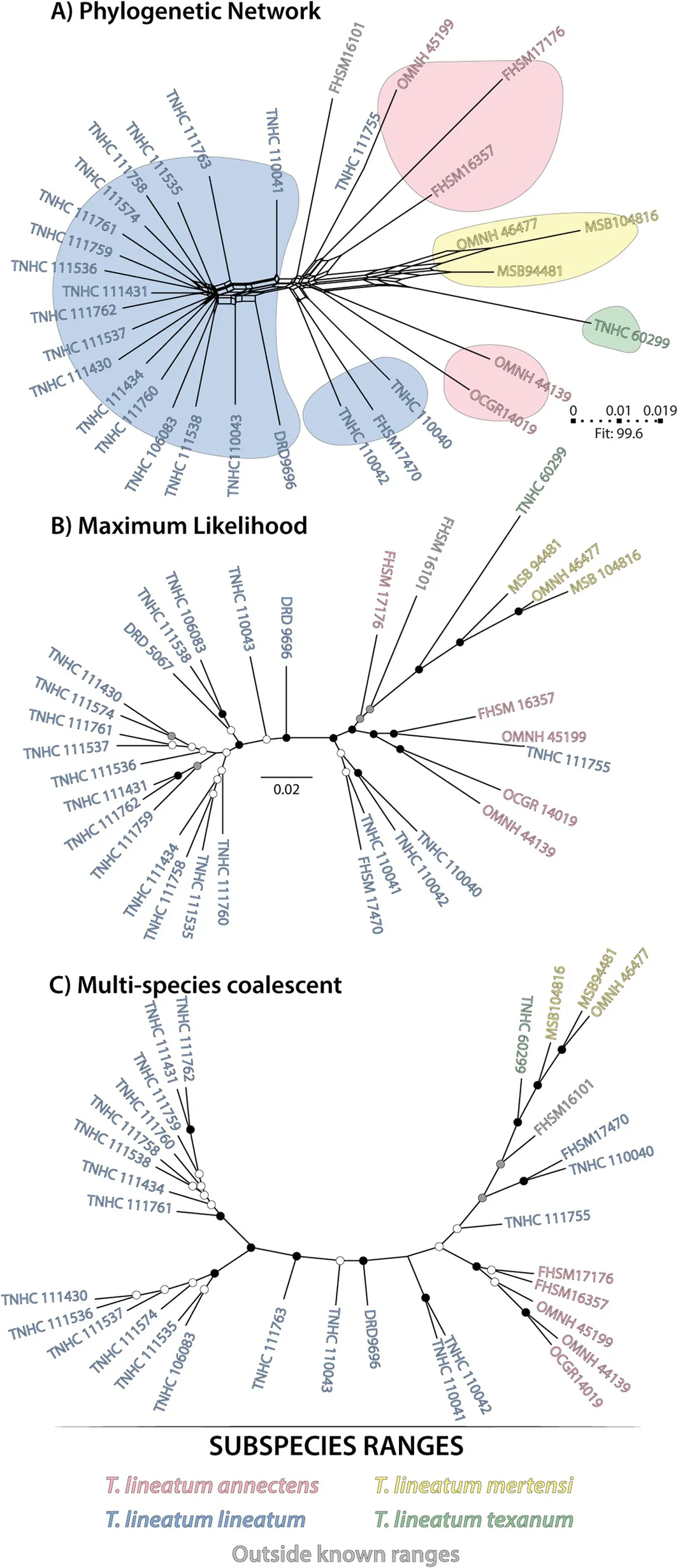
Phylogenetic relationships among samples inferred from the SNP dataset using (A) phylogenetic networks, (B) an unrooted maximum likelihood tree, and (C) an unrooted multispecies coalescent tree. Nodal support is indicated by circles representing bootstrap values: Black ≥ 90, gray ≥ 80, and white ≤ 79.

Similar topologies are recovered in both maximum likelihood (ML) and multispecies coalescent (MSC) unrooted trees (Figure 3B,C); however, support for specific clusters differs between methods. Under ML, *T. l. lineatum* forms a well‐supported cluster (excluding TNHC 111755), whereas this grouping is not supported under MSC and is incompatible with monophyly under any rooting (Figure 3C). Conversely, *T. l. annectens* forms a well‐supported cluster in the MSC tree but is not recovered in ML. However, in all three methods (phylogenetic networks, ML, and MSC) *T. l. mertensi* is recovered consistently as a distinct, well‐supported cluster. In addition, specimens from the James River population of South Dakota group together in a distinct cluster with high support across all topologies (Figure 3). Tests of isolation by distance reveal a strong positive correlation between pairwise genetic and geographic distance (*R* = 0.863, *p* = 0.001; Figure 4).

**FIGURE 4:**
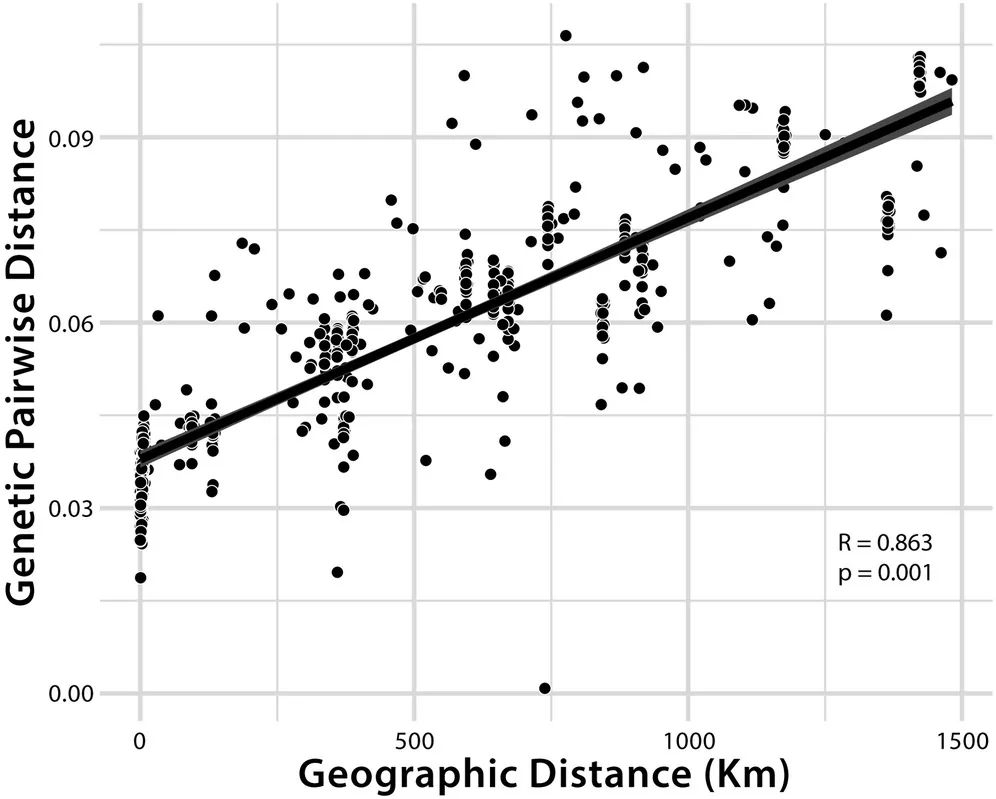
Scatter plot showing the relationship between pairwise genetic and geographic distances among individuals. The line of best fit is shown with a shaded 95% confidence interval. The Mantel statistic and associated *p*‐value are reported in the lower right‐hand corner of the plot. Table 1. Table of morphological characters used to delimit subspecies in their original descriptions. Both sexes are represented for each subspecies because the number of caudal scales was found to be sexually dimorphic. For counts of ventral and caudal scales the mean (*M*), standard deviation (SD), and range (R) are given.

The estimated *N*_e_ for the James River population was found to be 163.7 for the 85%, biallelic dataset not accounting for linkage disequilibrium, consistent across all minor allele frequency thresholds, with a parametric CI of 162.8–164.6. The jackknife CI however was much more variable, ranging from 22 to infinity, a common problem when working with small sample sizes (Mamoozadeh et al. 2025). Filtering the dataset to a single SNP per locus resulted in a larger estimate of *N*_e_ (426.5; parametric CI of 368.1–506.6, jackknife CI of 61 to infinity). Given the high uncertainty as evident by the jackknife confidence intervals, and that only 15 individuals were sampled, these results should be interpreted with caution. The observed heterozygosity (*H*_o_) of the James River population and the core population were very similar, with the James River population (*H*_o_ = 0.077; 0.100 using one SNP per locus) having only slightly reduced *H*_o_ relative to the core (*H*_o_ = 0.087; 0.116 using one SNP per locus). The *F*_ST_ value between the James River population and all other samples was 0.097 (0.103 using one SNP per locus), indicating moderate differentiation very recent population isolation (Hartl and Clark 1997).

## 4 Discussion

Our combined analyses indicate that population genetic structure within *T. lineatum* may be better characterized as clinal rather than partitioned into multiple discrete units (Figures 1, 2, 3, 4). Although sampling across the southern end of the range is sparse relative to the northern edge, the continuity of the distribution (Figure 4), linear isolation by distance, and lack of genetic structure are consistent with weak genetic differentiation and substantial connectivity. Signatures of range wide genetic connectivity across central North America have been detected in other widespread species of amphibians and reptiles, such as North American ground snakes (genus *Sonora*; Cox and Chippindale 2014), North American narrow‐mouthed toads (genus *Gastrophryne*; Streicher et al. 2012), and prairie rattlesnakes ( *Crotalus viridis* ; Smith et al. 2023). Such patterns may reflect the relatively continuous landscapes of the North American Great Plains, which lack more significant topographic barriers and may facilitate dispersal and gene flow across large spatial scales (Cox and Chippindale 2014).

How subspecies are defined, their value in taxonomy, and their acceptance and use across fields of study have been heavily debated in the literature (Burbrink et al. 2022; Guedes et al. 2026). Nevertheless, the subspecies of *T. lineatum* described by Ramsey (1953) and Smith (1965) appear to follow the historical precedent of using variation across a suite of phenotypic traits—often clinal, non‐discrete, or partially overlapping—as a proxy for evolutionary divergence and to identify geographically structured populations within a species (Meikle 1957; Phillimore et al. 2007; Mayr 1982). During the period in which Ramsey and Smith described these subspecies (the 1950s–1960s), the use of the subspecies rank (as defined by the number of annual subspecies descriptions) reached its peak (Uetz and Stylianou 2018), followed by a substantial decline from the latter half of the 20th century into the 21st century. Because modern population genetic and phylogenetic methods were not available at the time, these subspecific designations were necessarily based primarily on relatively limited morphological characters and may have reflected misconstrued patterns of intraspecific variation. As such, our phylogenetic analyses bring the validity of at least two of the four subspecies into question, *T. l. lineatum* and *T. l. annectens* (Figure 3), with support for both groups depending on the method of phylogenetic inference. Topological discordance can arise from violations of model assumptions, underlying evolutionary processes, and gene flow (Edwards et al. 2016; Leaché et al. 2014; Mirarab et al. 2021). For example, our concatenated ML analyses assume a single underlying topology across loci and therefore do not account for gene tree heterogeneity arising from processes such as incomplete lineage sorting (ILS) (Edwards et al. 2016; Leaché and Oaks 2017; Liu et al. 2015). In contrast, the MSC explicitly models gene tree discordance expected under ILS by estimating the species tree from a distribution of gene trees (Chifman and Kubatko 2014; Degnan and Rosenberg 2009; Jiang et al. 2020; Mirarab et al. 2021). Alternatively, discordance and weak support for relationships is an expected outcome of gene flow between populations (Leaché et al. 2014, 2023), in this case specifically between the subspecies of *T. l. annectens* and *T. l. lineatum* as supported by our phylogenetic network (Figure 3A). Thus, mixed phylogenetic signals may reflect substantial genetic connectivity across the range. While *T. l. mertensi* is consistently recovered as a well‐supported cluster across all phylogenetic methods, substantial sampling gaps in western Kansas, Oklahoma, and Texas limit our ability to draw strong inferences regarding its taxonomic status. Furthermore, *T. l. texanum* is represented by a single sample, further constraining interpretation. Because of these limitations, increased range‐wide sampling is needed in future studies, particularly targeting under sampled regions, with appropriate outgroup taxa incorporated for rooting phylogenetic analyses (i.e., *Regina grahamii* or other Thamnophiini species).

Given molecular evidence weakens support for the interpretation of the four recognized subspecies as independent, geographically structured populations, it seems pertinent to re‐examine the morphological characters used for their delimitation. In his 1953 treatment of *Tropidoclonion*, Ramsey's recognition of three distinct subspecies (*T. l. annectens, T. l. lineatum*, *and T. l. texanum*) and Smith's subsequent 1965 description of *T. lineatum mertensi* from New Mexico were based primarily on differences in ventral and caudal scalation, all of which show some overlap between subspecies (Table 1). The number of ventral scales differs from the other subspecies in *T. l. annectens* and *T. l. mertensi*, being higher in the former and lower in the latter across both sexes (Table 1). Furthermore, males of *T. l. texanum* had significantly lower caudal counts when compared to every other subspecies. However, Ramsey (1953) noted that 26 specimens appeared to be intergrades between *T. l. annectens* and *T. l. lineatum*, and 37 specimens were intergrades between *T. l. annectens* and *T. l. texanum* with males of intermediate caudal counts, in regions where their ranges intersect (Figure 1). Furthermore, Ramsey (1953) recovered subtle north–south geographic gradients in multiple characters, including the ventral, caudal, infralabial, and supralabial scale counts, dorsal scale row number, and body size. Although there may be true regional variation in these meristic and mensural characters, the existence of intermediate forms and molecular signals for continuous gene flow across the range suggest these characters may be clinal rather than discrete. Given the concordant patterns in both genetic and morphological data, together with the species' largely continuous distribution, we recommend future studies re‐examine the taxonomic validity of the four subspecies of *T. lineatum* .

**TABLE 1:** Table of morphological characters used to delimit subspecies in their original descriptions.

Subspecies	Sex (Sample size)	Ventral scale count	Caudal scale count	Source
*T. l. lineatum*	Male (32) Female (35)	*M* = 139.2; SD = 2.68; *R* = 135–148 *M* = 141.2; SD = 2.73; *R* = 135–148	*M* = 42.3; SD = 2.36; *R* = 34–47 *M* = 34.6; SD = 2.52; *R* = 31–40	Ramsey (1953)
*T. l. annectens*	Male (92) Female (118)	*M* = 145.4; SD = 3.00; *R* = 139–153 *M* = 147.0; SD = 2.54; *R* = 142–156	*M* = 42.4; SD = 2.38; *R* = 36–47 *M* = 34.3; SD = 1.93; *R* = 24–39	Ramsey (1953)
*T. l. texanum*	Male (44) Female (55)	*M* = 139.9; SD = 4.41; *R* = 132–150 *M* = 142.0; SD = 2.89; *R* = 136–148	*M* = 38.9; SD = 2.56; *R* = 34–45 *M* = 31.4; SD = 2.16; *R* = 25–36	Ramsey (1953)
*T. l. mertensi*	Male (22) Female (17)	*M* = 136.6; *R* = 131–142 *M* = 136.6; *R* = 134–141	*M* = 42.1; *R* = 39–47 *M* = 32.8; *R* = 30–47	Smith (1965)

*Note:* Both sexes are represented for each subspecies because the number of caudal scales was found to be sexually dimorphic. For counts of ventral and caudal scales, the mean (*M*), standard deviation (SD), and range (R) are given.

Although the validity of the recognized subspecies is uncertain, individuals from the South Dakota James River may represent a population that has become geographically isolated relatively recently (Figure 1). Our limited sampling across the species' distribution prevents a comprehensive comparison of genetic diversity and variation; however, we observed comparable, although lower, levels of heterozygosity *H*_o_ in the James River population relative to core populations. These results are somewhat surprising, given the general assumption that genetically divergent peripheral populations exhibit decreased genetic diversity (Brown et al. 1996; Lesica and Allendorf 1995; Vucetich and Waite 2003). One possible explanation for the moderately low *F*_st_ and comparable *H*_o_ to the core population is recent isolation following colonization, with fluctuating environmental conditions at the range edge possibly enhancing genetic drift and differentiation (Lammi et al. 1999; Sexton et al. 2009; Slatkin and Excoffier 2012). While this hypothesis remains to be tested in other small fossorial snakes, the James River population may represent a particularly promising system to investigate these dynamics.

There is growing recognition of the importance of conservation efforts for populations at the range edge (Channell 2005; Lesica and Allendorf 1995; Peterman et al. 2013), and the James River population may represent one such conservation priority. Its potential recent isolation, putatively small effective population size, and state endangered status in South Dakota (South Dakota Game, Fish, and Parks 2024) underscore its conservation significance. We recommend continued conservation efforts focused on habitat protection and long‐term monitoring of the James River population. Additionally, two seemingly disjunct populations, one in the Denver, Colorado region and another centered around St. Louis, Missouri and extending into Illinois (Figure 1), are not represented in our dataset (Ramsey 1953; Smith and Smith 1963; Stebbins 2003). Dense, range‐wide sampling, particularly incorporating these relict populations, will be necessary to fully characterize genetic diversity and population structure in *T. lineatum* and to inform future conservation management decisions.

## Author Contributions

**Taylor Probst:** conceptualization (equal), data curation (lead), formal analysis (lead), investigation (lead), methodology (lead), project administration (supporting), software (lead), validation (lead), visualization (lead), writing – original draft (lead), writing – review and editing (lead). **Cameron D. Siler:** conceptualization (equal), funding acquisition (equal), project administration (lead), resources (lead), supervision (equal), validation (equal), visualization (equal), writing – review and editing (supporting). **Drew R. Davis:** conceptualization (lead), funding acquisition (equal), project administration (lead), resources (equal), supervision (equal), writing – review and editing (supporting).

## Funding

Funding was provided, in part, by an award from the South Dakota Game, Fish, and Parks Wildlife Diversity Small Grants Program to D.R.D., and by the University of Oklahoma and Oklahoma Biological Survey.

## Conflicts of Interest

The authors declare no conflicts of interest.

## Data Availability

Raw demultiplexed sequencing reads are available from the NCBI (National Center for Biotechnology Information) Sequence Read Archive (SRA) under BioProject ID: PRJNA1506669 http://www.ncbi.nlm.nih.gov/bioproject/1506669. Processed alignments and files necessary for de‐multiplexing the dataset as performed here are available at Figshare DOI: https://doi.org/10.6084/m9.figshare.33386947.

## Appendix 1 Data Associated With Lined Snake ( *Tropidoclonion lineatum* ) Tissue Samples Analyzed in This Project

**Table:**

Museum #	Field #	State	County	Latitude	Longitude	Date collected	Sequenced successfully?
TNHC 110043	DRD 1464	South Dakota	Minnehaha	43.58782	−96.64719	21 Sept 2014	Yes
TNHC 106083	DRD 4468	South Dakota	Hutchinson	43.44341	−97.73601	20 Oct 2017	Yes
TNHC 111535	DRD 4699	South Dakota	Hutchinson	43.43038	−97.77872	30 April 2018	Yes
TNHC 111574	DRD 4702	South Dakota	Hutchinson	43.42972	−97.79432	28 April 2018	Yes
TNHC 111430	DRD 4703	South Dakota	Hutchinson	43.43005	−97.79426	28 April 2018	Yes
TNHC 111536	DRD 4704	South Dakota	Hutchinson	43.43045	−97.79340	30 April 2018	Yes
TNHC 111537	DRD 4705	South Dakota	Hutchinson	43.43043	−97.79379	1 May 2018	Yes
TNHC 111431	DRD 4706	South Dakota	Hutchinson	43.43043	−97.79379	1 May 2018	Yes
TNHC 111434	DRD 4707	South Dakota	Hutchinson	43.43042	−97.79380	1 May 2018	Yes
TNHC 111538	DRD 4724	South Dakota	Hutchinson	43.42413	−97.83804	2 May 2018	Yes
TNHC 111758	DRD 5062	South Dakota	Hutchinson	43.44320	−97.76244	3 Oct 2018	Yes
TNHC 111759	DRD 5063	South Dakota	Hutchinson	43.44320	−97.76244	3 Oct 2018	Yes
TNHC 111760	DRD 5064	South Dakota	Hutchinson	43.45087	−97.77903	3 Oct 2018	Yes
TNHC 111761	DRD 5065	South Dakota	Hutchinson	43.43223	−97.77884	3 Oct 2018	Yes
TNHC 111762	DRD 5066	South Dakota	Hutchinson	43.42793	−97.78735	3 Oct 2018	Yes
TNHC 111763	DRD 5067	South Dakota	Hutchinson	43.42692	−97.83805	3 Oct 2018	Yes
TNHC 111755	DRD 5068	South Dakota	Hutchinson	43.38605	−97.75151	3 Oct 2018	Yes^a^
TNHC 111756	DRD 5069	South Dakota	Hutchinson	43.38594	−97.75397	3 Oct 2018	No
—	DRD 9696	Minnesota	Rock	43.69553	−96.19023	5 Sept 2022	Yes
FHSM 17470	—	Nebraska	Fillmore	40.42298	−97.39482	15 Oct 2016	Yes
TNHC 110040	DRD 3797	Nebraska	Gage	40.09172	−96.50165	15 Oct 2016	Yes
TNHC 110041	DRD 3798	Nebraska	Gage	40.21677	−96.59545	15 Oct 2016	Yes
TNHC 110042	DRD 3799	Nebraska	Gage	40.33695	−96.53932	15 Oct 2016	Yes
FHSM 16582	—	Nebraska	Hall	40.79526	−98.45672	29 Aug 2014	No
FHSM 16357	—	Kansas	Neosho	37.70331	−95.30188	28 April 2008	Yes
FHSM 16101	—	Kansas	Scott	38.65620	−100.97300	29 July 2011	Yes
FHSM 17176	—	Kansas	Sedgwick	37.64600	−97.45500	5 Feb 2015	Yes
OMNH 46477	—	Oklahoma	Cimarron	36.95736	−102.96247	7 Oct 2017	Yes
OMNH 44139	—	Oklahoma	Cleveland	35.20630	−97.41180	20 April 2014	Yes
OCGR 14019	JLW 050	Oklahoma	Oklahoma	35.48765	−97.50433	6 Jan 2009	Yes
OMNH 45199	—	Oklahoma	Osage	36.84576	−96.29875	21 May 2014	Yes
MSB 104816	—	New Mexico	Bernalillo	35.16182	−106.28651	5 Aug 2021	Yes
MSB 78416	BLC 820	New Mexico	Lincoln	33.56884	−105.51412	20 Aug 2010	No
MSB 94481	MTH 611	New Mexico	Otero	32.75000	−105.47008	17 May 2012	Yes
MSB 76556	MSB Field 7607	New Mexico	Torrance	34.35406	−105.66695	3 July 2009	No
TNHC 60299	TJL 372	Texas	Schleicher	30.85311	−100.60774	29 Aug 1998	Yes

Abbreviations: DRD, Drew R. Davis Field Series; FHSM, Sternberg Museum of Natural History, Fort Hays State University; MSB, Museum of Southwestern Biology, University of New Mexico; OCGR, Oklahoma Center for Genomic Resources; OMNH, Sam Noble Oklahoma Museum of Natural History; TJL, Travis J. LaDuc Field Series; TNHC, Biodiversity Collections, The University of Texas at Austin.

^a^Sample had a high percentage of missing data (80.7%) of the final concatenated loci dataset.

## Appendix 2 Summary Statistics for Sequencing, Read Filtering, and Loci Assembly of Successful Samples Used in This Study

**Table:**

Sample ID	Raw read #	# Reads passing filters	Total clusters #	Clusters depth	Est. heterozygosity	Est. error	Consensus reads #	Loci in assembly #
TNHC 110043	4,249,752	4,058,689	188,585	81,514	0.007868	0.00118	72,862	58,640
TNHC 110040	6,333,272	6,097,391	238,760	109,424	0.006584	0.001117	98,367	79,216
TNHC 110041	6,843,740	6,546,935	205,178	89,330	0.007317	0.000886	82,654	67,370
TNHC 110042	4,701,723	4,380,118	183,357	72,841	0.008071	0.001054	65,343	53,515
TNHC 106083	10,524,529	10,104,327	290,224	117,885	0.008317	0.001075	104,248	82,387
TNHC 111535	6,098,851	5,913,294	246,130	117,361	0.00655	0.001151	105,300	84,190
TNHC 111574	978,789	922,131	133,492	48,382	0.009721	0.001579	44,948	38,516
TNHC 111430	15,860,866	15,130,402	367,795	116,884	0.013012	0.001076	102,399	79,721
TNHC 111536	23,062,601	21,819,422	481,393	122,105	0.015009	0.001092	102,761	79,251
TNHC 111537	16,710,677	16,024,905	421,830	127,884	0.013797	0.001181	110,313	83,555
TNHC 111431	8,244,551	7,698,966	256,120	83,039	0.014116	0.000918	75,926	61,227
TNHC 111434	38,047,463	35,680,802	633,546	119,821	0.018173	0.001275	94,385	71,146
TNHC 111538	28,180,014	27,017,602	610,428	137,215	0.013701	0.001253	112,960	82,574
TNHC 111758	20,773,262	19,797,491	428,311	99,284	0.018516	0.001071	87,018	65,460
TNHC 111759	8,119,037	7,440,437	274,402	67,943	0.013466	0.001068	61,010	49,811
TNHC 111760	8,095,368	7,789,163	236,591	110,404	0.007239	0.001019	99,103	78,619
TNHC 111761	11,965,761	11,476,871	322,835	121,021	0.00914	0.001011	106,838	84,690
TNHC 111762	1,587,542	1,527,511	145,182	55,627	0.008088	0.001032	52,677	44,761
TNHC 111763	9,512,812	9,192,843	250,597	120,011	0.006771	0.001055	106,314	84,316
TNHC 111755	13,854,340	13,256,863	368,533	127,046	0.010962	0.001134	110,799	84,789
DRD 9696	4,526,306	4,358,009	184,720	100,818	0.006263	0.000922	93,932	75,231
FHSM 16101	1,494,667	1,305,404	122,090	39,305	0.009893	0.001794	37,141	32,269
FHSM 16357	5,513,013	5,229,018	354,167	105,096	0.009507	0.001186	94,786	66,949
FHSM 17176	15,241,091	14,569,822	307,627	118,428	0.009221	0.001056	102,119	80,215
FHSM 17470	10,027,705	9,483,556	311,286	108,292	0.009523	0.00104	96,365	76,399
MSB 104816	4,502,548	4,330,493	192,281	90,007	0.007245	0.00111	83,887	67,465
MSB 94481	20,531,254	19,617,685	409,932	127,980	0.00987	0.001156	109,923	83,292
OMNH 44139	16,612,756	15,882,299	449,263	138,786	0.011402	0.000914	122,112	86,932
OCGR 14019	12,433,569	11,842,917	299,144	113,960	0.009677	0.001021	100,726	80,330
OMNH 45199	14,886,259	14,252,019	373,701	133,738	0.008545	0.001025	117,005	90,733
OMNH 46477	18,043,656	17,427,788	308,426	125,292	0.010093	0.000878	113,288	86,630
TJL 372	6,794,959	6,498,516	227,456	102,952	0.006768	0.001194	92,894	73,330
